# Practical considerations for evaluation of images of the breast during pregnancy and lactation

**DOI:** 10.1590/0100-3984.2021.54.1e2

**Published:** 2021

**Authors:** Norma Maranhão, Beatriz Maranhão

**Affiliations:** 1 Member of the National Mammography Commission of the Colégio Brasileiro de Radiologia e Diagnóstico por Imagem (CBR), Director of Lucilo Maranhão Diagnósticos, Recife, PE, Brazil. E-mail: normamaranhao@hotmail.com.br.; 2 Instituto de Medicina Integral Prof. Fernando Figueira (IMIP), Director of Lucilo Maranhão Diagnósticos, Recife, PE, Brazil. E-mail: beatrizmaranhao@lucilomaranhao.com.br.

The physiological changes that occur during the pregnancy-postpartum period, including breast changes, represent a diagnostic challenge, given that they result in specific characteristics^(1,2)^. Therefore, it is crucial that radiologists understand those changes. A review article published in the previous issue of **Radiologia Brasileira** addresses such physiological changes, the safety of breast imaging methods, and the main findings of breast diseases that occur during that period^(3)^.

The physical examination of the breasts is impaired by increases in breast volume and firmness, as well as by greater numbers of breast nodules, resulting from hormonal stimuli^(4)^, whereas an increase in breast density, particularly in young women, makes it technically more difficult to assess the breast in imaging examinations^(5)^, because it can mimic certain diseases, as well as confusing the assessment of other pre-existing diseases^(6)^. Therefore, changes in the breast during these physiological states can delay the diagnosis of carcinoma, although breast cancer is less common in women who are pregnant or lactating than in those of the same age who are not.

Pregnancy-related breast cancer comprises cases discovered during pregnancy or up to one year after delivery^(6)^. It usually exhibits biological behavior that is more aggressive than that of other forms of breast cancer, and its diagnosis is typically delayed because of the physiological changes that occur during pregnancy, underestimation of the signs/symptoms reported by the patient^(7)^, and difficulty in interpreting imaging examinations.

Ultrasound was the main imaging method employed in the evaluation of palpable lesions and in the search for malignant lesions. Mammography, albeit less sensitive, mainly because of the high density of the breast parenchyma^(8)^, proved to be useful for the evaluation of lesions considered suspicious in the clinical or ultrasound examination, with a minimal risk of exposing the fetus to ionizing radiation. In lactating patients, mammography should preferably be performed after breastfeeding or pumping, in order to decrease breast density^(9)^.

The use of magnetic resonance imaging (MRI) has been avoided during the gestational period, taking into account the passage of gadolinium into the amniotic fluid. During the lactation period, MRI is useful in the evaluation of some specific lesions. However, due to the increased vascularization of breast tissue during that period, it is less accurate in the evaluation of pregnancy-related breast cancer, which makes it useful as a complementary tool. In the case of detectable carcinomas, the MRI findings of pregnancy-related breast cancer do not differ from those of cancer unrelated to pregnancy^(8)^.

Percutaneous biopsies, preferably guided by ultrasound, can be performed normally during pregnancy and lactation, although the risk of complications inherent to the procedure is theoretically increased in pregnant and lactating women, because of increased vascularization of the breast parenchyma, milk production, ductal dilation, and the breast trauma inherent to breastfeeding^(8)^. However, it is possible to take certain measures to reduce the number of complications.

Most of the palpable breast changes during pregnancy and lactation will be diagnosed as benign disease, some of which, such as lactation adenoma and galactocele, are specific to the period, galactocele being the most common benign mass in lactating women^(4)^, typically seen after the cessation of breastfeeding^(1)^ and regressing spontaneously thereafter.

Lactational adenoma is a benign tumor related to physiological changes in the pregnancy-lactation period, occurring most commonly during the third trimester of pregnancy and during lactation^(4)^. The main differential diagnosis of lactational adenoma is fibroadenoma, the distinction being made on the basis of the fact that adenomas regress spontaneously after pregnancy or cessation of lactation^(6)^. The two can be indistinguishable on imaging examinations, the typical ultrasound presentation being a BI-RADS category 3 lesion.

Fibroadenoma is the most common benign breast tumor in the pregnancy-lactation period, in most cases being present prior to pregnancy, and its identification is facilitated by the volume increase resulting from the increase in hormone levels and regression after the cessation of breastfeeding^(6)^. The typical imaging findings of fibroadenoma in the pregnancy-lactation period are similar to those of fibroadenoma in women who are non-pregnant and non-lactating, potentially containing cystic areas resulting from secretory hyperplasia and changes during lactation, with milk accumulation mimicking a galactocele.

Mastitis is an inflammatory process of the breast, resulting from infection or other conditions, and the most common complication is abscess, defined as a purulent collection. Although mastitis is rare during pregnancy, it is relatively common during lactation, occurring in approximately 10% of lactating women, usually within the first six weeks after delivery^(10)^, due to nipple cracks/fissures, obstruction/engorgement of the milk ducts, and inadequate milk drainage^(8)^. The diagnosis is based on clinical findings, imaging examinations (preferably ultrasound) being reserved for complicated cases in which an abscess is suspected and for cases that are refractory to treatment^(4)^.

Idiopathic granulomatous mastitis is a rare, chronic inflammatory condition of the breast that is common in women who have been pregnant and have lactated, usually occurring within the first six years after pregnancy^(11)^. Although an autoimmune hypothesis has been proposed, the ultimate cause remains unknown. Clinical and radiological manifestations are variable and nonspecific, sometimes being suggestive of malignancy, making the diagnosis of idiopathic granulomatous mastitis a major challenge^(11)^. Therefore, the pathological study is decisive, showing lobular non-caseating granulomas^(11)^ and allowing the differential diagnosis to be made with other diseases, such as tuberculosis, fungal infections, sarcoidosis, and Wegener’s granulomatosis.

In conclusion, for all of the reasons presented above, it is necessary to have an adequate understanding of the physiological changes and benign breast lesions common in the pregnancy-lactation period, in order to differentiate such changes from pregnancy-related breast cancer. Thus, diagnostic delays can be avoided, enabling a safe, satisfactory approach and increasing the effectiveness of treatment.
